# Chronic kidney disease with comorbid cardiac dysfunction exacerbates cardiac and renal damage†

**DOI:** 10.1111/jcmm.13349

**Published:** 2017-10-10

**Authors:** Shan Liu, Bing H. Wang, Darren J. Kelly, Henry Krum, Andrew R. Kompa

**Affiliations:** ^1^ School of Medicine South China University of Technology China; ^2^ Centre of Cardiovascular Research and Education in Therapeutics Department of Epidemiology and Preventive Medicine Monash University Melbourne Australia; ^3^ Department of Medicine St. Vincent's Hospital University of Melbourne Melbourne Australia

**Keywords:** chronic renocardiac syndrome, subtotal nephrectomy, myocardial infarction, cardiac remodelling, renal tubulointerstitial fibrosis

## Abstract

To address the pathophysiological mechanisms underlying chronic kidney disease with comorbid cardiac dysfunction, we investigated renal and cardiac, functional and structural damage when myocardial infarction (MI) was applied in the setting of kidney injury (induced by 5/6 nephrectomy—STNx). STNx or Sham surgery was induced in male Sprague–Dawley rats with MI or Sham surgery performed 4 weeks later. Rats were maintained for a further 8 weeks. Rats (*n* = 36) were randomized into four groups: Sham+Sham, Sham+MI, STNx+Sham and STNx+MI. Increased renal tubulointerstitial fibrosis (*P* < 0.01) and kidney injury molecule‐1 expression (*P* < 0.01) was observed in STNx+MI compared to STNx+Sham animals, while there were no further reductions in renal function. Heart weight was increased in STNx+MI compared to STNx+Sham or Sham+MI animals (*P* < 0.05), despite no difference in blood pressure. STNx+MI rats demonstrated greater cardiomyocyte cross‐sectional area and increased cardiac interstitial fibrosis compared to either STNx+Sham (*P* < 0.01) or Sham+MI (*P* < 0.01) animals which was accompanied by an increase in diastolic dysfunction. These changes were associated with increases in ANP, cTGF and collagen I gene expression and phospho‐p38 MAPK and phospho‐p44/42 MAPK protein expression in the left ventricle. Addition of MI accelerated STNx‐induced structural damage but failed to significantly exacerbate renal dysfunction. These findings highlight the bidirectional response in this model known to occur in cardiorenal syndrome (CRS) and provide a useful model for examining potential therapies for CRS.

## Introduction

The classification and definition of CRS was proposed by Ronco *et al*. in 2008 1 and re‐evaluated by the Acute Dialysis Quality Initiative (ADQI) in 2013 2. CRS has promoted increased attention but has also generated much debate regarding a more precise definition, and a mechanistic framework enabling appropriate design of clinical and experimental studies. For the time being the classification of Ronco *et al*. (2008) has been widely adopted 1.

Patients with chronic kidney disease (CKD) are at extreme high cardiovascular risk. Acceleration of coronary artery disease and left ventricular hypertrophy (LVH) are major cardiac complications observed in renal failure that may be contributory to heart failure, frequently accompanied in CKD 3. Mortality rate among patients with end‐stage renal disease (ESRD) remains above 20% per annum, with more than half of deaths related to cardiovascular events 4.

In order to understand the process and mechanisms involved in type 4 CRS, CKD leading to chronic heart failure (CHF), studies have attempted to develop suitable animal models. Initially models were established using a uni‐nephrectomy model of CKD followed by CHF, induced by myocardial infarction‐MI. These studies did not recapitulate the phenomenon of primary renal failure as renal function was preserved in these animals 5, 6, 7, 8. LVH and hypertension is a consistent feature observed in early CKD patients, severe hypertension was not a feature in the uni‐nephrectomy‐MI model. Utilizing a more severe model of CKD induced by 5/6 nephrectomy (STNx) followed 2 weeks later by MI, Windt *et al*. (2008) observed compromised cardiac function did not lead to reduced renal function and increased structural changes in the kidney when compared to animals with STNx alone at 12 weeks 9. The negative findings and the absence of sufficient cardiac assessment left many pathophysiological questions unanswered. Bongartz *et al*. (2012) speculated that failure to observe worsening renal function or injury may be due to the short duration of CKD prior to MI; hence, STNx duration was prolonged to 8 weeks. Although renal function was similar in STNx+MI compared STNx alone, glomerulosclerosis had worsened 10. Cardiac changes in STNx+MI animals were associated with diastolic dysfunction and aggravated cardiac dilation compared with MI alone 10. Exploration of mechanisms in chronic renocardiac syndrome relies on animal models; however, very few animal studies have investigated renal and cardiac changes in the setting of CKD with comorbid cardiac dysfunction; furthermore, the existing studies have not reached a consensus 11.

This study aimed to further investigate the molecular, structural and functional changes in heart and kidney in this model (STNx+MI), and place these findings in context with previous renocardiac models. Furthermore, in an attempt to determine whether failure of the primary organ in CRS results in different outcomes, we compared our previous findings examining the effects of MI complicated by the addition of STNx (MI+STNx) 12 with the current model (STNx+MI).

## Materials and methods

### Study design and rat model

**Figure 1:**
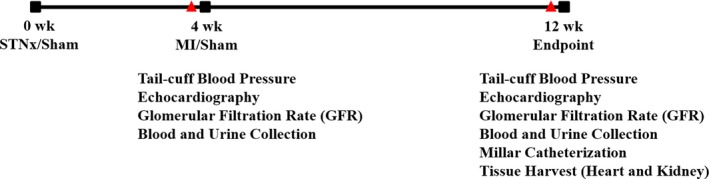
Experimental design. STNx, 5/6 nephrectomy; MI, myocardial infarction.

Male Sprague–Dawley rats (*n* = 36) weighing 200–250 g were randomized into four groups: (*i*) Sham‐operated STNx + Sham‐operated MI (Sham+Sham), (*ii*) Sham‐operated STNx + MI (Sham+MI), (*iii*) STNx + Sham‐operated MI (STNx+Sham) (*iv*) and STNx + MI. STNx or Sham surgery was initially performed, 4 weeks later MI or Sham surgery was induced (Fig. 1). Systolic blood pressure (BP) was measured in conscious rats using tail cuff plethysmography prior to MI/Sham surgery and 8 weeks later prior to the end of the study. Similarly, renal and cardiac function was assessed prior to the MI/Sham surgery and prior to the end of the study. Hemodynamic parameters were measured prior to sacrifice and tissues collected for analysis. Kaplan–Meier survival analysis was performed in GraphPad Prism 5 (GraphPad Software Inc. La Jolla, CA, USA). All experiments adhered to the Guidelines for Animal Welfare of the National Health and Medical Research Council of Australia and approved by the Ethics Committee of St Vincent's Hospital (AEC approval number: 027/10).

### Surgery

STNx surgery was performed as previously described 12. Isoflurane‐anaesthetized rats underwent right subcapsular nephrectomy and infarction of approximately 2/3 of the left kidney, assessed by visible blanching, by selective ligation of two of 3–4 extra‐renal branches of the left renal artery. Sham‐operated STNx rats underwent laparotomy and manipulation of both kidneys before wound closure. STNx/Sham rats underwent either MI or Sham‐operated MI as previously described at week 4 12. Isoflurane anaesthetized rats were intubated and a thoracotomy performed. The left anterior descending coronary artery was identified and permanently ligated 3 mm below its origin. Buprenorphine (0.03 mg/kg, sc) was administered for analgesia. Sham‐operated MI rats underwent the same procedure except that the coronary artery was not ligated.

### Glomerular filtration rate

Glomerular filtration rate (GFR) was assessed prior to MI/Sham surgery and again at the end‐point as previously described 12. Intravenous administration of ^99^ technetium‐diethylene triamine penta‐acetic acid (^99^Tc‐DTPA) was followed by blood sampling 43 min. after injection and plasma radioactivity measured and compared to the counts of the standard reference prepared at the time of injection. The calculated GFR was corrected for body weight (BW) and reported as ml/min./kg.

### Creatinine clearance and proteinuria

Prior to the MI/Sham surgery and at the end‐point, rats were bled and then housed in metabolic cages for urine collection. Serum creatinine, urinary creatinine and proteinuria were measured as previously described using a Cobas Integra® 400 Plus Bioanalyzer (Roche, Indianapolis, IN, USA) 12.

### Echocardiography

Transthoracic echocardiography was performed as previously described prior to MI/Sham surgery and at the end‐point using a Vivid 7 (GE Vingmed, Horten, Norway) echocardiography machine with a 10‐MHz phased array probe 12. This procedure is routinely performed in our laboratory to assess systolic and diastolic function 13.

### Cardiac catheterization

Hemodynamic measurements were assessed prior to tissue collection 12. Briefly, anaesthetized rats were intubated and ventilated, and a 2F miniaturized combined catheter/micromanometer (Model SPR838 Millar instruments, Houston, TX, USA) was inserted into the right common carotid artery to obtain aortic BP and then advanced into the LV to obtain LV pressure–volume (PV) loops. PV loops were recorded at steady state and during transient preload reduction, achieved by occlusion of the inferior *vena cava* and portal vein with the ventilator turned off and the rat apnoeic. The following parameters were assessed using Millar conductance data acquisition and analysis software PVAN 3.2: LV end diastolic pressure (LVEDP); the maximal rate of pressure rise (dP/dtmax) and fall (dP/dtmin); the slope of the end systolic PV relationship (ESPVR); the slope of the end diastolic PV relationship (EDPVR); Tau Logistic; and the slope of preload recruitable stroke work relationship (PRSW).

### Histology and immunohistochemistry

LV and kidney tissues were fixed in 10% neutral buffered formalin and then processed for histopathology. Paraffin‐embedded sections (4 μm) were prepared for histological staining. Sections were stained with picrosirius red and scanned using Aperio ScanScope Console (v.8.0.0.1058 Aperio Technologies, Inc., Vista, CA, USA) as previously described 12. Infarct size was expressed as an averaged percentage of the endocardial and epicardial scarred circumferences of the LV. Small infarcts (<20%) were omitted from the analysis. Picrosirius red stained interstitial fibrosis in the non‐infarct zone of the LV and kidney, excluding perivascular fibrosis, was selected for its intensity of red staining, and the percentage area was calculated using a pre‐set algorithm 12. The intensity and algorithm was pre‐set and maintained constant for the analysis of all sections.

Myocyte hypertrophy was determined from haematoxylin–eosin stained sections and scanned using Aperio ScanScope Console to determine myocyte cross‐sectional area. Myocytes in the same plain were assessed by selecting cells with similar sized nuclei and intact cellular membranes, these were outlined and the average calculated from 50 myocytes per LV 12.

Antibody staining of LV sections for collagen I (Southern Biotech, Birmingham, AL, USA) and collagen III (BioGenex, San Ramon, CA, USA), kidney injury molecule‐1 (KIM‐1; R&D Systems, Minneapolis, MN, USA) and macrophage infiltration (CD68; AbD Serotec, Raleigh, NC, USA) was assessed by detection with diaminobenzidene as previously described 12. Images were digitally captured using an AxioImager.A1 microscope (Carl Zeiss AxioVision, North Ryde, NSW, Australia.) attached to an AxioCamMRc5 digital camera (Carl Zeiss AxioVision). Positive staining for collagen I, III and KIM‐1 was quantified using image analysis software AIS (Analytic Imaging Station version 6.0, Imaging Research Inc., Ontario, Canada) selected for its intensity, and results were expressed as percentage area calculated using a pre‐set algorithm. Positive macrophage staining (CD68 immunoreactive cells) was counted in whole tissue sections. All histological and immunohistochemical data were acquired and analysed by a single blinded observer.

### Quantitative mRNA expression

Total RNA extracted from frozen LV non‐infarct tissue and kidney tissue (RNAqueous^®^ Kit, Ambion, Austin, TX, USA) was reverse transcribed to cDNA. Triplicate cDNA aliquots were amplified using sequence‐specific primers (Geneworks, Adelaide, SA, Australia) with SYBR Green detection (Applied Biosystems, Foster City, CA, USA) using an ABI prism 7900HT sequence detection system (Applied Biosystems). Messenger RNA expression was quantified for transforming growth factor (TGF) β_1_, connective tissue growth factor (cTGF), collagen I, collagen IV, atrial natriuretic peptide (ANP), β‐myosin heavy chain (β‐MHC) and IL‐6. Quantitation was standardized to the housekeeping genes GAPDH (cardiac tissue) and 18S (renal tissue). The primer pair sequences are as previously described 12.

### Western blot analysis

Total protein was extracted from frozen LV non‐infarct tissue and kidney tissue with modified RIPA buffer containing protease and phosphatase inhibitors. Protein concentrations were determined by Bradford assay (Bio‐Rad, Hercules, CA, USA), and Western blot analysis was performed as described previously with specific antibodies (phospho‐p38 mitogen‐activated protein kinase (MAPK), phospho‐p44/42 MAPK, phospho‐nuclear factor kappa B (NFκB) and TGF‐β antibodies (Cell Signaling Technology, Beverly, MA, USA; pan‐actin antibody‐NeoMarkers, Fremont, CA, USA) 12.

### Statistical analyses

All statistical analyses were performed using GraphPad Prism 5. Results are expressed as the mean ± S.E.M. Significance was determined by a two‐way anova with Student–Neuman–Keuls post hoc test *P* < 0.05. Pooled data in Table 6 were analysed by a one‐way anova with Bonferroni's multiple comparison test. For comparisons between 2 groups, unpaired Student's *t*‐test was used, *P* < 0.05 are considered statistically significant.

## Results

### Survival rate, infarct size, tissue weight and blood pressure

**Figure 2:**
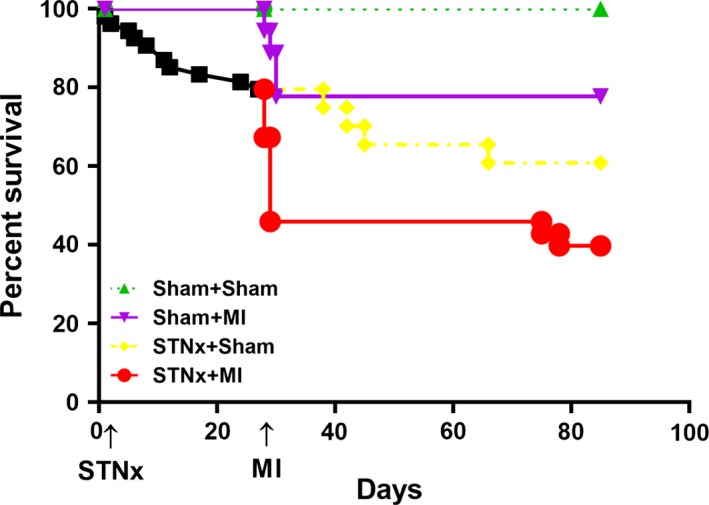
Kaplan–Meier curves for groups of Sham+Sham, Sham+MI, STNx+Sham and STNx+MI, respectively. To discriminate from long‐term mortality, the decrease in survival at day 28–30 post‐MI indicates acute post‐operative mortality.

Survival rate at 12 weeks was 100%, 77.8%, 60.9% and 39.8% in Sham+Sham, Sham+MI, STNx+Sham and STNx+MI groups, respectively (Fig. 2). The number of surviving rats in each group at the end‐point is shown in Table 1. In the sham‐operated STNx group, there was 100% survival at 4 weeks, of those the 11 underwent MI, four died within 2 days (36.4% acute post‐operative mortality); no further mortality was observed in this group over the length of the study. Of the 46 rats that underwent STNx surgery, 10 died before the second surgery. In the STNX+Sham group, of the 16 rats assigned, four died (25%), whereas in the STNx‐MI group, of the 20 rats that underwent MI, 11 died within 2 days (55% acute post‐operative mortality), of the remaining nine rats, two died prior to study conclusion (22.2% long‐term mortality). No difference in infarct size was observed in STNx+MI *versus* Sham+MI rats (Table 1).

**Table 1:** Animal number, survival rate, infarct size and tissue weight. Values are mean ± S.E.M

	Sham+Sham *N* = 10	Sham+MI *N* = 7	STNx+Sham *N* = 12	STNx+MI *N* = 7
Survival rate (%)	100.0	77.8%	60.9%	39.8%
Infarct size (%)	–	36.3 ± 3.6	–	34.4 ± 2.4
Tissue weight				
Bodyweight (g)	547.8 ± 10.9	537.7 ± 14.1	459.7 ± 7.8***,§§	440.4 ± 26.3§§§
Heart weight/BW ratio (mg/g)	2.43 ± 0.03	2.90 ± 0.11	3.74 ± 0.12***,§	4.57 ± 0.44§§§,#
Lung weight/BW ratio (mg/g)	2.87 ± 0.17	3.73 ± 0.54	3.74 ± 0.07	4.10 ± 0.41
LV weight/BW ratio (mg/g)	1.69 ± 0.02	1.98 ± 0.10	2.85 ± 0.11***,§§	3.41 ± 0.40§§§
Right ventricle weight/BW ratio (mg/g)	0.49 ± 0.01	0.60 ± 0.04*	0.54 ± 0.02	0.72 ± 0.04§,###
Atria weight/BW ratio (mg/g)	0.23 ± 0.01	0.33 ± 0.04	0.35 ± 0.01*	0.44 ± 0.07
Left kidney weight/BW ratio (mg/g)	3.09 ± 0.08	3.22 ± 0.07	4.32 ± 0.11***,§§§	4.77 ± 0.22§§§

BW, bodyweight.

*P < 0.05, ***P < 0.001 *versus* Sham+Sham; ^§^ *P* < 0.05, ^§§^^§^ *P* < 0.001 *versus* Sham+MI.; ^#^ *P* < 0.05, ^#^^#^^#^ *P* < 0.001 *versus* STNx+Sham.

The STNx+MI group had significantly lower bodyweight than the Sham+MI group (*P* < 0.001); however, no difference was observed compared to STNx+Sham animals (Table 1). All tissue weights were calculated as a ratio of bodyweight. Heart weight (*P* < 0.001), LV weight (*P* < 0.001) and right ventricle weight (*P* < 0.05) were increased in STNx+MI rats compared to the Sham+MI group (Table 1). A major driver of this increase in hypertrophy was the associated increase in tail cuff blood pressure (BP) (*P* < 0.001; Table 2). No difference in BP was observed in STNx+MI *versus* STNx+Sham rats. Heart weight (*P* < 0.05) and right ventricle weight (*P* < 0.001) were significantly higher in STNx+MI *versus* STNx+ Sham rats.

**Table 2:** Blood pressure and echocardiography data at week 4 (prior to MI/Sham surgery) and week 12

	Week 4				Week 12			
	Sham+Sham *N*=10	Sham+MI *N* = 7	STNx+Sham *N*=12	STNx+MI *N* = 7	Sham+Sham *N*=10	Sham+MI *N* = 7	STNx+Sham *N* = 12	STNx+MI *N* = 7
Blood pressure (mmHg)	120.3 ± 5.7	117.9 ± 3.0	193.3 ± 6.2***,§§§	178.3 ± 6.7§§§	138.3 ± 4.4	130.1 ± 8.8	221.8 ± 9.1***,§§§	204.9 ± 11.6§§§
Echocardiography								
FS (%)	41.4 ± 0.7	39.1 ± 1.5	45.8 ± 1.3*,§§	47.1 ± 2.0§§	40.0 ± 1.2	16.5 ± 0.9***	43.8 ± 2.6§§§	18.1 ± 1.2###
Anterior wall thickness (mm)	1.37 ± 0.01	1.32 ± 0.02	1.84 ± 0.06***,§§§	1.83 ± 0.05§§§	1.56 ± 0.03	0.78 ± 0.02***	2.14 ± 0.07***,§§§	0.82 ± 0.01###
Posterior wall thickness (mm)	1.70 ± 0.05	1.60 ± 0.04	1.97 ± 0.07*	2.12 ± 0.12§§§	1.72 ± 0.04	1.85 ± 0.06	2.26 ± 0.09***,§§	2.38 ± 0.12§§
Relative wall thickness	0.39 ± 0.01	0.36 ± 0.01	0.47 ± 0.03**,§§	0.49 ± 0.03§§	0.37 ± 0.01	0.32 ± 0.01***	0.52 ± 0.03*,§§§	0.43 ± 0.03§, ##
LVEF (%)	68.8 ± 2.1	66.3 ± 3.1	74.3 ± 2.0**,§§	79.2 ± 3.0§§§	66.9 ± 1.9	42.0 ± 2.4***	74.3 ± 2.7*,§§§	42.7 ± 3.5##
LVEDV (ml)	0.54 ± 0.04	0.56 ± 0.03	0.50 ± 0.03	0.54 ± 0.05	0.81 ± 0.06	1.14 ± 0.04**	0.65 ± 0.04*,§§§	0.97 ± 0.10##
LVESV (ml)	0.17 ± 0.02	0.19 ± 0.02	0.13 ± 0.01§	0.11 ± 0.02§	0.25 ± 0.02	0.66 ± 0.04***	0.18 ± 0.03§§§	0.55 ± 0.07###
LV mass (gram/m^2^)	1.42 ± 0.02	1.40 ± 0.01	1.62 ± 0.04**,§§	1.77 ± 0.08§§§,#	1.6 ± 0.02	1.72 ± 0.07	1.95 ± 0.08**	2.0 ± 0.16
DT (msec)	29.7 ± 1.0	30.3 ± 0.9	33.2 ± 0.6*	33.4 ± 1.2	28.8 ± 0.7	34.7 ± 1.0**	39.0 ± 1.0***,§	37.6 ± 1.9
IVRT (msec)	21.1 ± 0.9	20.2 ± 1.2	24.0 ± 1.2	24.2 ± 0.9	23.0 ± 1.2	29.2 ± 1.6**	28.4 ± 1.6*	29.7 ± 2.1
E wave velocity (m/sec.)	1.14 ± 0.03	1.08 ± 0.06	1.05 ± 0.03	1.14 ± 0.06	1.08 ± 0.02	1.12 ± 0.08	1.0 ± 0.04	1.04 ± 0.11
A wave velocity (m/sec.)	0.53 ± 0.03	0.54 ± 0.05	0.75 ± 0.05**,§§	0.83 ± 0.05§§§	0.45 ± 0.03	0.31 ± 0.04	0.81 ± 0.05***,§§§	0.51 ± 0.06§,###
E’ wave velocity (cm/sec.)	5.2 ± 0.3	4.8 ± 0.3	4.1 ± 0.2*	4.5 ± 0.3	4.8 ± 0.2	4.5 ± 0.3	3.5 ± 0.3*	3.9 ± 0.5
A’ wave velocity (cm/sec.)	2.9 ± 0.2	2.7 ± 0.2	3.8 ± 0.2**,§§	4.7 ± 0.3§§§,##	2.4 ± 0.2	1.9 ± 0.2	4.2 ± 0.3***,§§§	3.3 ± 0.2§§§,#
E/E’ wave ratio	22.6 ± 1.2	23.1 ± 1.8	25.8 ± 1.1	25.8 ± 1.8	23.3 ± 1.4	25.9 ± 2.7	30.0 ± 2.1	28.9 ± 5.3
E/A wave ratio	2.2 ± 0.1	2.1 ± 0.1	1.5 ± 0.1***,§§	1.4 ± 0.1§§	2.5 ± 0.2	4.1 ± 0.7*	1.3 ± 0.1***^,§§§^	2.5 ± 0.7§
E’/A’ wave ratio	1.8 ± 0.1	1.9 ± 0.2	1.1 ± 0.1***,§§§	1.0 ± 0.1§§§	2.1 ± 0.2	2.4 ± 0.2	0.9 ± 0.1***,§§§	1.2 ± 0.2§§§

Values are mean ± S.E.M.

*P < 0.05, **P < 0.01, ***P < 0.001 *versus* Sham+Sham; ^§^ *P* < 0.05, ^§§^ *P* < 0.01, ^§§^^§^ *P* < 0.001 *versus* Sham+MI; ^#^ *P* < 0.05, ^#^^#^ *P* < 0.01, ^#^^#^^#^ *P* < 0.001 *versus* STNx+Sham.

FS, fractional shortening; LVEF, left ventricular ejection fraction; LVEDV and LVESV, left ventricular end diastolic and end systolic volume; DT, deceleration time; IVRT, isovolumetric relaxation time.

### Cardiac function

Left ventricular ejection fraction (LVEF), fractional shortening (FS) and ESPVR were reduced in STNx+MI *versus* STNx+Sham rats (*P* < 0.01) at 12 weeks. LV end diastolic volume (LVEDV) and end systolic volume (LVESV) were significantly increased in STNx+MI animals compared to STNx+Sham (*P* < 0.01; Table 2 and 3). Systolic dysfunction was not further exaggerated in STNx+MI compared to Sham+MI animals.

**Table 3:** Hemodynamic parameters assessed at week 12

	Sham+Sham *N* = 10	Sham+MI *N* = 7	STNx+Sham *N* = 12	STNx+MI *N* = 7
Heart rate (beats/min.)	297 ± 21	316 ± 10	312 ± 19	320 ± 27
dP/dtmax (mmHg/msec)	6306 ± 138	4478 ± 182***	6589 ± 295§§§	5667 ± 255§§
−dP/dtmin (mmHg/msec)	5479 ± 314	3192 ± 161**	4315 ± 396*	3605 ± 348
LVEDP (mmHg)	2.6 ± 0.6	8.8 ± 1.4**	5.7 ± 1.1	8.8 ± 1.8
ESPVR (mmHg/μl)	0.47 ± 0.07	0.25 ± 0.03	0.89 ± 0.12**^,§§^	0.38 ± 0.03##
EDPVR (mmHg/μl)	0.013 ± 0.002	0.055 ± 0.011***	0.037 ± 0.006**	0.045 ± 0.007
PRSW (mmHg)	84.2 ± 6.3	42.9 ± 6.7**	80.4 ± 8.0§§	62.0 ± 11.4
Tau logistic (msec)	11.4 ± 0.6	13.0 ± 0.8	17.6 ± 1.2***,§	17.0 ± 1.6§

Values are mean ± S.E.M.

*P < 0.05, **P < 0.01, ***P < 0.001 *versus* Sham+Sham; ^§^ *P* < 0.05, ^§§^ *P* < 0.01 *versus* Sham+MI; ^#^^#^ *P* < 0.01 *versus* STNx+Sham.

dp/dtmax and dp/dtmin, the maximal rate of pressure rise and fall; LVEDP, left ventricular end diastolic pressure; ESPVR and EDPVR, slope of end systolic and diastolic pressure‐volume relationship; PRSW, slope of preload recruitable stroke work relationship.

Diastolic function measured at week 12 saw a reduction in E/A and E’/A’ ratio and an increase in A’ and A wave velocity in both STNx groups *versus* Sham+MI rats (*P* < 0.05). Deceleration time (DT) and EDPVR were significantly increased in Sham+MI and STNx+Sham rats compared to the control Sham+Sham group (*P* < 0.01). Tau logistic, was prolonged in both STNx groups *versus* Sham+MI rats at week 12 (*P* < 0.05; Tables 2 and 3). Diastolic dysfunction was not augmented in the STNx+MI compared to the STNx+Sham group.

### Cardiac interstitial fibrosis

**Figure 3:**
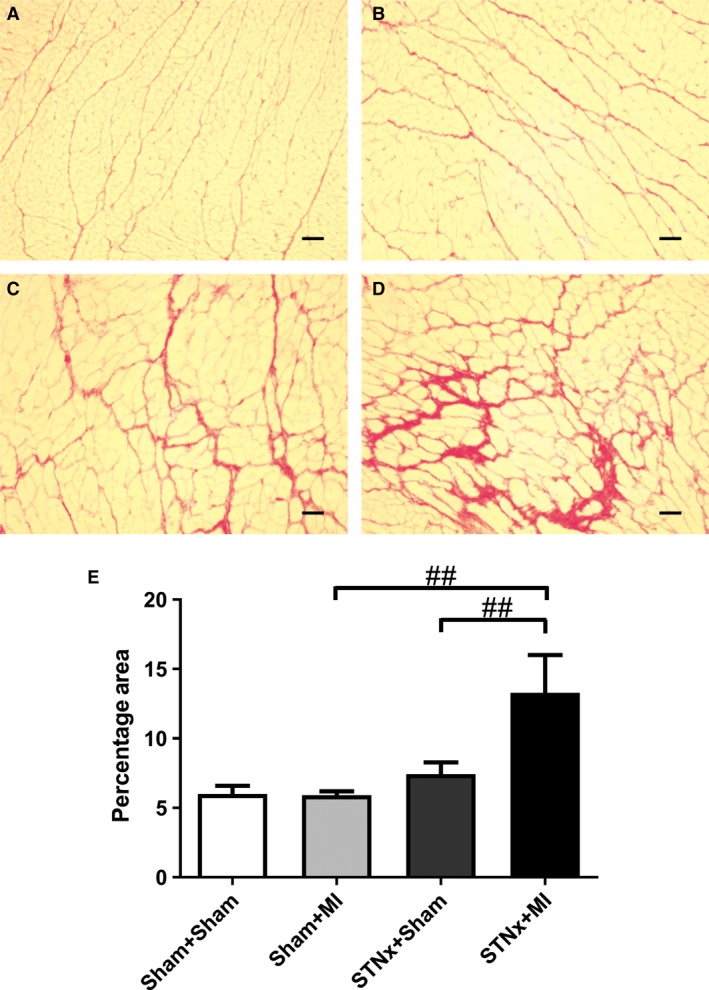
Representative images of the LV non‐infarct zone showing picrosirus red staining from the following groups (**A**) Sham+Sham, (**B**) Sham+MI, (**C**) STNx+Sham and (**D**) STNx+MI. Scale bar, 100 µm. Quantitation of picrosirius red staining (**E**) showing STNx+MI rats had greater cardiac interstitial fibrosis compared to Sham+MI and STNx+Sham groups. Data are expressed as mean ± S.E.M. ^##^ *P* < 0.01 for between group comparisons.

**Figure 4:**
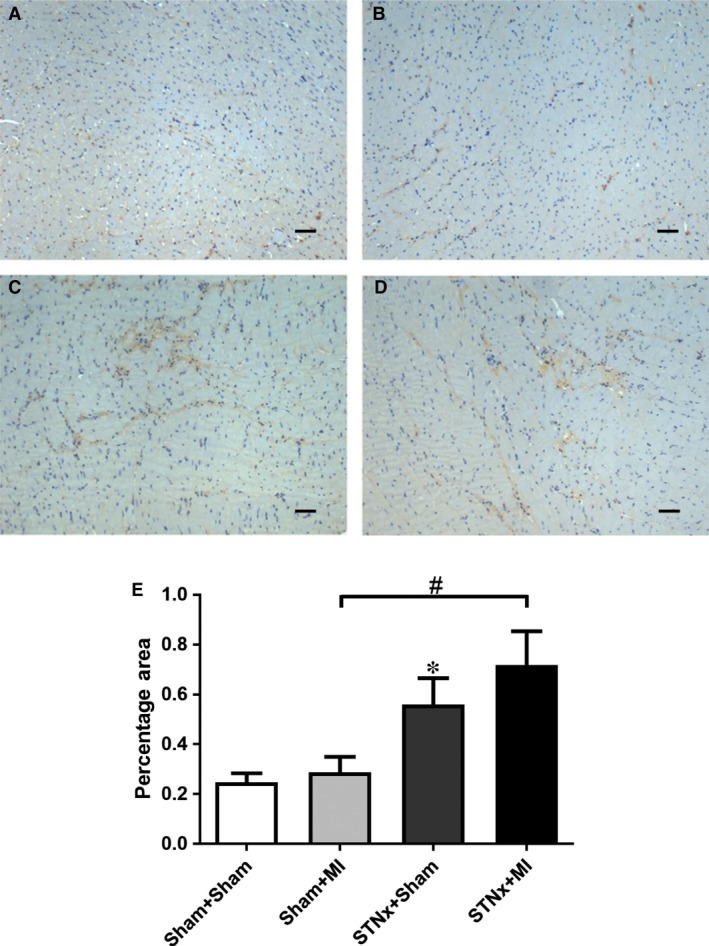
Representative images of the LV non‐infarct zone showing collagen I immunostaining from the following groups (**A**) Sham+Sham, (**B**) Sham+MI, (**C**) STNx+Sham and (**D**) STNx+MI. Scale bar, 30 µm. Quantitation of collagen I (**E**) showing STNx+MI rats had greater immunostaining compared to Sham+MI group. Data are expressed as mean ± S.E.M. *P < 0.05 *versus* Sham+Sham; ^#^ *P* < 0.05 for between group comparisons.

**Figure 5:**
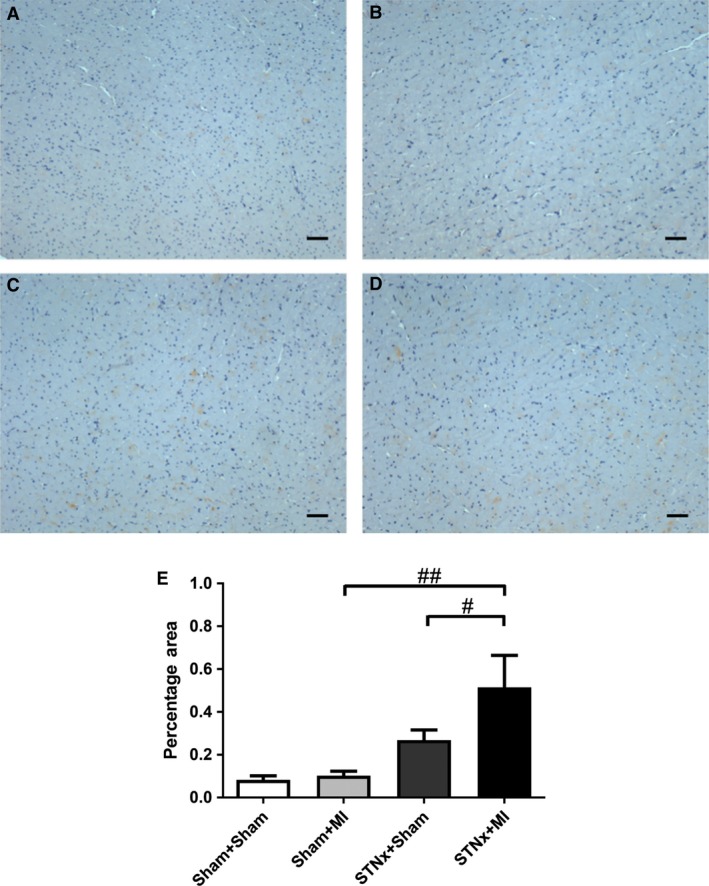
Representative images of the LV non‐infarct zone showing collagen III immunostaining from the following groups (**A**) Sham+Sham, (**B**) Sham+MI, (**C**) STNx+Sham and (**D**) STNx+MI. Scale bar, 30 µm. Quantitation of collagen III (**E**) showing STNx+MI rats had greater immunostaining than the Sham+MI and STNx+Sham groups. Data are expressed as mean ± S.E.M. ^#^ *P* < 0.05, ^##^ *P* < 0.01 for between group comparisons.

Cardiac interstitial fibrosis was increased in STNx+MI rats compared to STNx+Sham (*P* < 0.01) and Sham+MI (*P* < 0.01) groups (Fig. 3). Cardiac collagen I and III was increased in STNx+MI rats compared to Sham+MI rats (*P* < 0.05; Figs 4 and 5). Collagen III was increased in STNx+MI rats compared to the STNx+Sham group (*P* < 0.05).

### Cardiac hypertrophy

**Figure 6:**
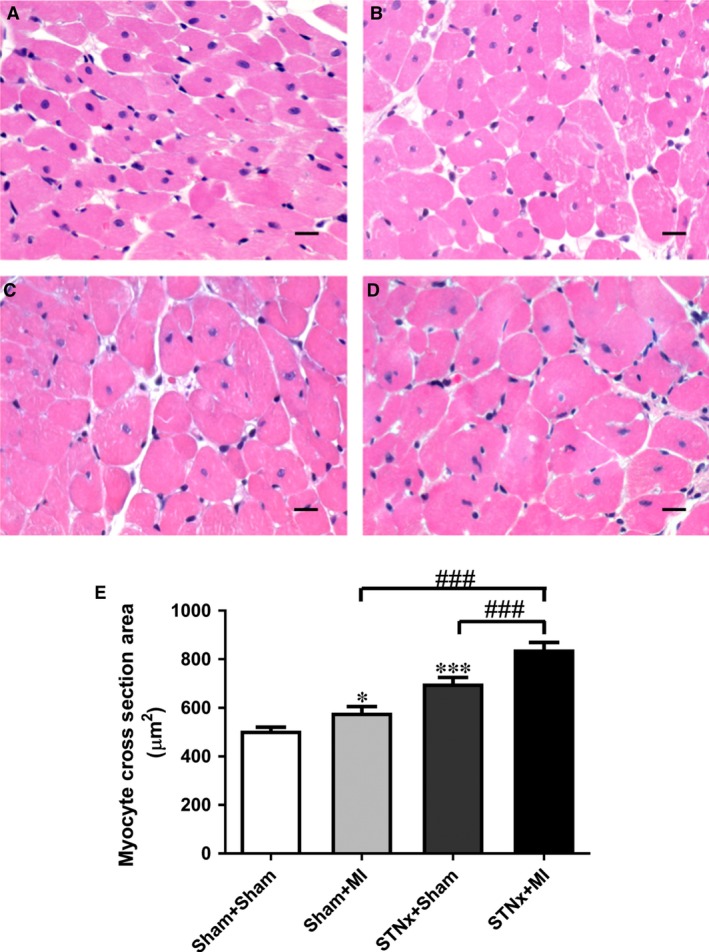
Representative images of the LV non‐infarct zone showing haematoxylin and eosin staining in myocytes from the following groups (**A**) Sham+Sham, (**B**) Sham+MI, (**C**) STNx+Sham and (**D**) STNx+MI. Scale bar, 30 µm. Quantitation of the data (**E**) showing cardiomyocyte cross‐sectional area was increased in STNx+MI animals compared to Sham+MI and STNx+Sham groups. Data are expressed as mean ± S.E.M. *P < 0.05, ***P < 0.001 *versus* Sham+Sham; ^###^ *P* < 0.001 for between group comparisons.

Heart weight and right ventricular weight were increased in STNx+MI animals compared to both STNx+Sham and Sham+MI groups (*P* < 0.05). Left ventricular weight was increased in STNx+MI *versus* Sham+MI groups (*P* < 0.001). Echocardiographic analysis demonstrated an increase in posterior wall thickness and relative wall thickness in STNx+MI *versus* Sham+MI (*P* < 0.05), and in STNx+Sham *versus* Sham+MI animals (*P* < 0.01). Myocyte cross‐sectional area was elevated in Sham+MI (*P* < 0.05) and STNx+Sham (*P* < 0.001) groups compared to the Sham+Sham group (Fig. 6). Myocyte cross‐sectional area was further increased in STNx+MI compared to both Sham+MI and STNx+Sham groups (*P* < 0.001).

### Cardiac mRNA expression

**Figure 7:**
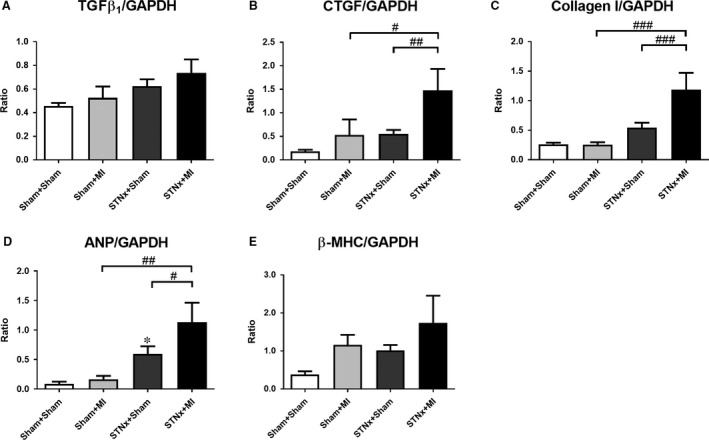
mRNA expression of pro‐fibrotic related markers TGFβ_1_ (**A**), cTGF (**B**) and collagen I (**C**) and hypertrophic related markers ANP (**D**) and β‐MHC (**E**), expressed as a ratio of GAPDH in the LV non‐infarct zone. Data are expressed as mean ± S.E.M. *P < 0.05 *versus* Sham+Sham; ^#^ *P* < 0.05, ^##^ *P* < 0.01, ^###^ *P* < 0.001 for between group comparisons.

Gene expression of the pro‐fibrotic‐related markers cTGF and collagen I, but not TGFβ_1_ was increased in the STNx+MI animals compared to the STNx+Sham (*P* < 0.01) and the Sham+MI groups (*P* < 0.05; Fig. 7A–C). The hypertrophic‐related marker ANP but not β‐MHC was increased in STNx+MI animals compared the STNx+Sham (*P* < 0.05) and the Sham+MI groups (*P* < 0.01; Fig. 7D–E). There was no difference in GADPH gene expression between the groups (Fig. S1A).

### Cardiac signalling pathway activation

**Figure 8:**
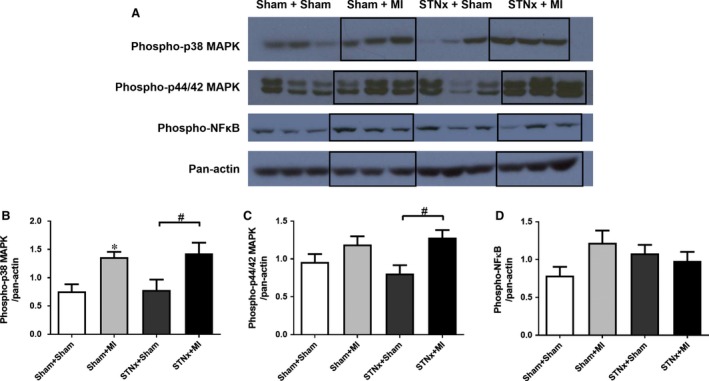
Representative images of the Western blot showing cardiac protein levels in the LV non‐infarct zone from Sham+Sham, Sham+MI, STNx+Sham and STNx+MI groups (**A**). Quantitation of protein levels for phospho‐p38 MAPK (**B**), phospho‐p44/42 MAPK (**C**), and phospho‐NFκB (**D**) was normalized with pan‐actin. Levels of phospho‐p38 MAPK and phospho‐p44/42 MAPK, but not phospho‐NFκB were increased in the LV in STNx+MI compared to the STNx+Sham group. Data are expressed as mean ± S.E.M. *P < 0.05 *versus* Sham+Sham; ^#^ *P* < 0.05 for between group comparisons.

STNx+MI animals demonstrated increased levels of phospho‐p38 MAPK (*P* < 0.05) and phospho‐p44/42 MAPK (*P* < 0.05) compared to the STNx+Sham group (Fig. 8). Activation of TGF‐β was not detectable in the LV tissues. No significant differences were observed in phospho‐NFκB between the groups.

### Renal function

STNx‐operated rats developed severe renal dysfunction at weeks 4 and 12, respectively (*P* < 0.001; Table 4). However, no further deterioration in renal function, as indicated by reduced GFR, reduced creatinine clearance and increased proteinuria, was observed in STNx+MI *versus* STNx+Sham rats at week 12.

**Table 4:** Renal function at week 4 (prior to MI/Sham surgery) and week 12

	Week 4				Week 12			
	Sham+Sham *N* = 10	Sham+MI *N* = 7	STNx+Sham *N* = 12	STNx+MI *N* = 7	Sham+Sham *N* = 10	Sham+MI *N* = 7	STNx+Sham *N* = 12	STNx+MI *N* = 7
Glomerular filtration rate (ml/min./kg)	9.9 ± 0.6	9.4 ± 0.7	2.5 ± 0.2***,§§§	3.3 ± 0.4§§§	8.6 ± 0.5	8.1 ± 0.5	0.9 ± 0.4***^,§§§^	0.4 ± 0.3§§§
Creatinine clearance (ml/min.)	232.9 ± 10.4	246.5 ± 17.0	36.9 ± 4.5***,§§§	45.1 ± 4.7§§§	242.6 ± 26.6	255.5 ± 39.7	32.2 ± 8.7***^,§§§^	33.9 ± 12.4§§§
Serum creatinine (μmol/L)	23.2 ± 0.9	22.8 ± 0.7	63.7 ± 3.2***,§§§	58.5 ± 1.9§§§	36.1 ± 5.2	29.0 ± 0.7	111.8 ± 15.6**,§§	145.0 ± 27.8§§
Proteinuria (mg/24 hrs)	20.3 ± 2.0	16.2 ± 3.3	76.7 ± 16.0**,§	75.9 ± 20.2§	23.1 ± 2.0	16.2 ± 1.6	344.4 ± 48.9***,§§§	380.0 ± 89.1§§§

Values are mean ± S.E.M.

**P < 0.01, ***P < 0.001 *versus* Sham+Sham; ^§^ *P* < 0.05, ^§§^ *P* < 0.01, ^§§^^§^ *P* < 0.001 *versus* Sham+MI.

### Renal tubulointerstitial fibrosis, inflammation and injury markers

**Figure 9:**
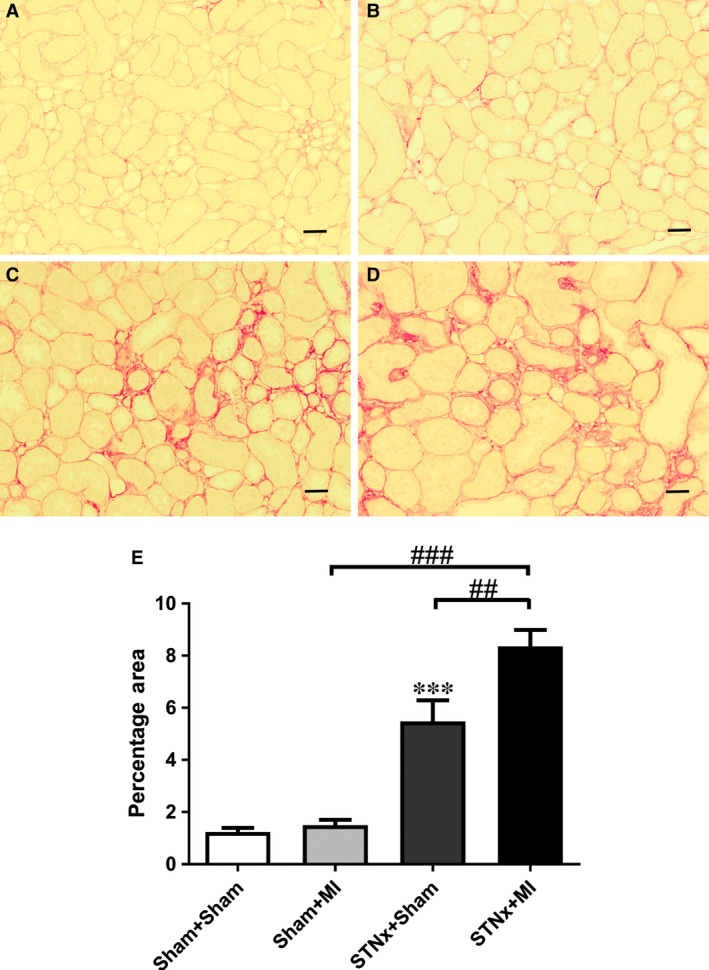
Representative images of the non‐infarct zone of the kidney showing picrosirus red staining from the following groups (**A**) Sham+Sham, (**B**) Sham+MI, (**C**) STNx+Sham and (**D**) STNx+MI. Scale bar, 50 µm. Quantitation of renal tubulointerstitial fibrosis (**E**) showing STNx+MI animals had greater tubulointerstitial fibrosis than Sham+MI and STNx+Sham groups. Data are expressed as mean ± S.E.M. ***P < 0.001 *versus* Sham+Sham; ^##^ *P* < 0.01, ^###^ *P* < 0.001 for between group comparisons.

**Figure 10:**
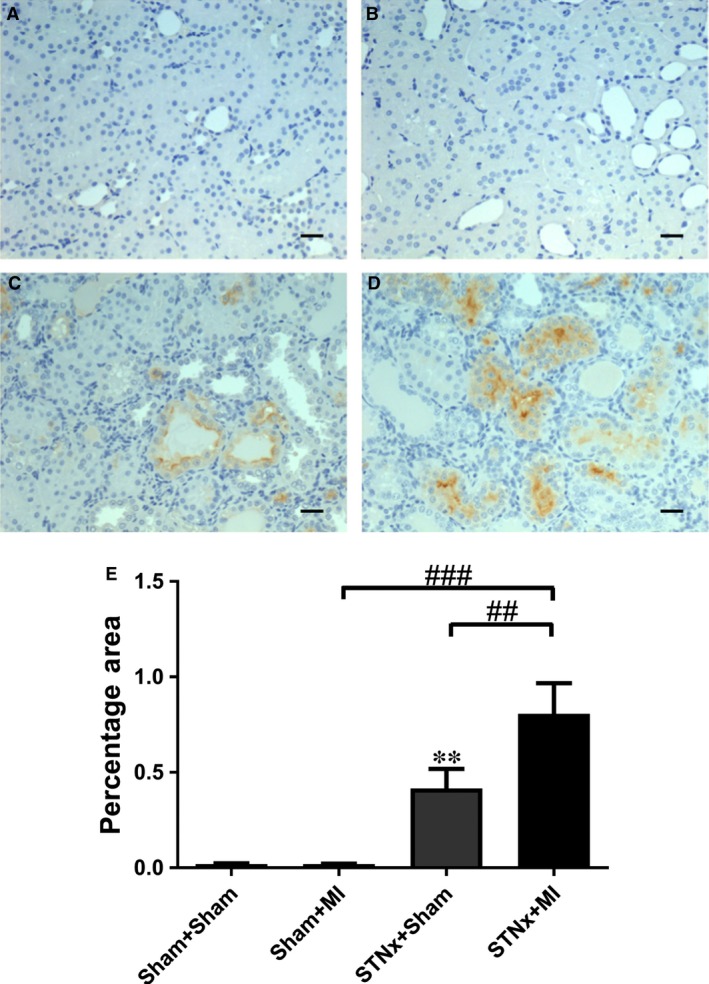
Representative images of the non‐infarct cortex region of the kidney showing immunostaining of KIM‐1 from the following groups (**A**) Sham+Sham, (**B**) Sham+MI, (**C**) STNx+Sham and (**D**) STNx+MI. Scale bar, 30 µm. Quantitation of KIM‐1 (**E**) showing STNx+MI animals had greater KIM‐1 expression compared to Sham+MI and STNx+Sham groups. Data are expressed as mean ± S.E.M. **P < 0.01 *versus* Sham+Sham; ^##^ *P* < 0.01, ^###^ *P* < 0.001 for between group comparisons.

**Figure 11:**
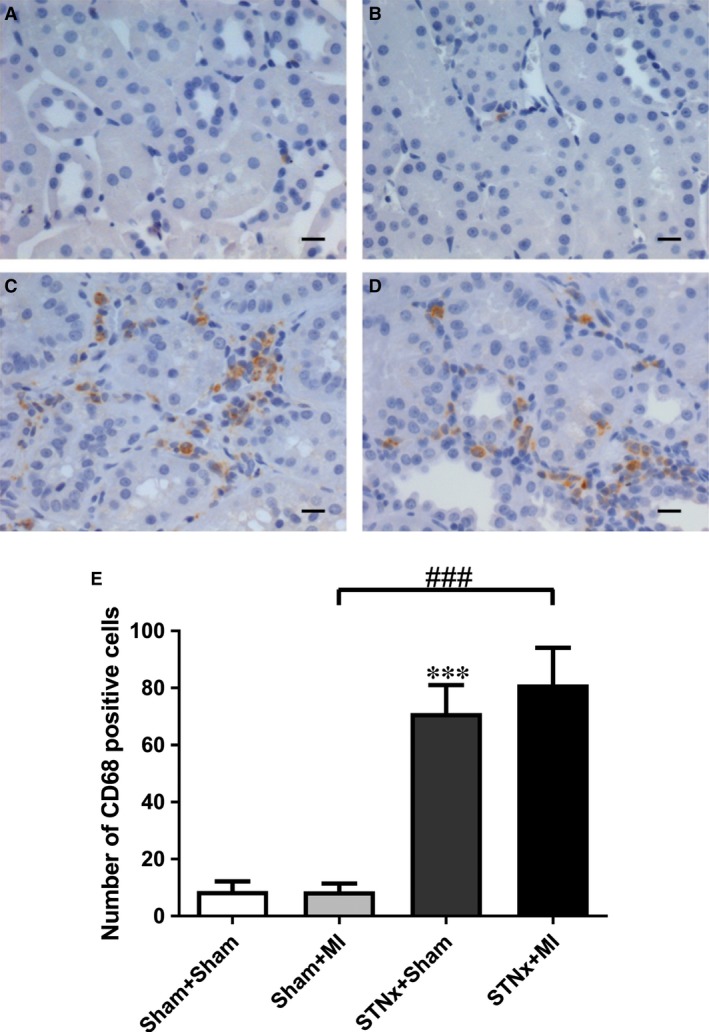
Representative images of the non‐infarct cortex region of the kidney showing immunostaining of macrophage infiltration from the following groups (**A**) Sham+Sham, (**B**) Sham+MI, (**C**) STNx+ Sham and (**D**) STNx+MI. Scale bar, 30 µm. Quantitation of the number of CD68 positive cells (**E**) showing STNx+MI animals had a greater number of macrophages than the Sham+MI group. Data are expressed as mean ± S.E.M. ***P < 0.001 *versus* Sham+Sham; ^###^ *P* < 0.001 for between group comparisons.

Renal fibrosis (*P* < 0.001), KIM‐1 expression (*P* < 0.01) and macrophage infiltration (*P* < 0.001) were increased in STNx+Sham *versus* Sham+Sham (Figs 9, 10, 11). STNx+MI animals demonstrated greater tubulointerstitial fibrosis and KIM‐1 expression compared to STNx+Sham (*P* < 0.01) and Sham+MI (*P* < 0.001) groups (Figs 9 and 10). Macrophage infiltration was increased in STNx+MI animals (*P* < 0.001) compared to the Sham+MI but not the STNx+Sham group (Fig. 11).

### Renal mRNA expression

**Figure 12:**
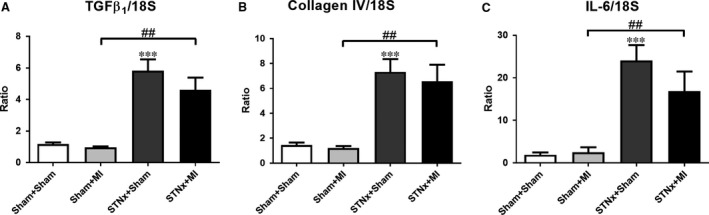
mRNA expression of pro‐fibrotic‐related markers TGFβ_1_ (**A**) and collagen IV (**B**); and the pro‐imflammatory cytokine 1L‐6 (**C**) in the kidney, expressed as a ratio of 18S. Data are expressed as mean ± S.E.M. ***P < 0.001 *versus* Sham+Sham; ^##^ *P* < 0.01 for between group comparisons.

Renal pro‐fibrotic‐related markers TGFβ_1_ and collagen IV as well as pro‐inflammatory cytokine IL‐6 were increased in STNx+MI *versus* Sham+MI animals (*P* < 0.01; Fig. 12); and no difference was observed between STNx+MI *versus* STNx+Sham groups. There was no difference in 18S gene expression between the groups (Fig. S1B).

### Renal signalling pathway activation

**Figure 13:**
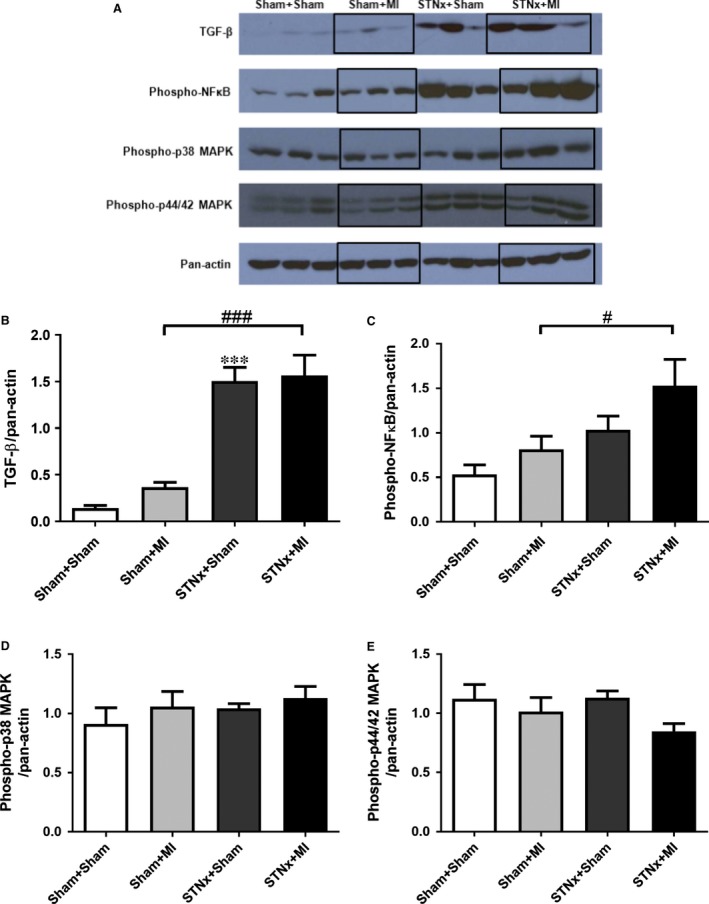
Representative images of the Western blot showing renal protein levels from Sham+Sham, Sham+MI, STNx+Sham and STNx+MI groups (**A**). Quantitation of the protein levels of TGF‐β (**B**), phospho‐NFκB (**C**), phospho‐p38 MAPK (**D**) and phospho‐p44/42 MAPK (**E**), as normalized with pan‐actin. Levels of TGF‐β and NFκB expression were increased in the kidney in STNx+MI compared to the Sham+MI group. Data are expressed as mean ± S.E.M. ***P < 0.001 *versus* Sham+Sham; ^#^ *P* < 0.05, ^###^ *P* < 0.001 for between group comparisons.

TGF‐β was increased in STNx+Sham (*P* < 0.001) compared to Sham+Sham animals (Fig. 13). Both STNx groups (*P* < 0.001) demonstrated increased levels of TGF‐β protein expression compared to the Sham+MI group. Phospho‐NFκB (*P* < 0.05) was elevated in STNx+MI *versus* Sham+MI animals. No significant differences were observed in phospho‐p38 MAPK and phospho‐p44/42 MAPK between the groups.

## Discussion

The present study established a model of renocardiac syndrome where initial renal injury was followed by MI. This is a potentially useful model to examine the pathophysiology and mechanisms underlying CRS. Previous studies 9, 10 have examined a similar model; however, MI initiation was at different time‐points post‐STNx, at 2 weeks 9 and 8 weeks 10 with similar parameters measured. Therefore, we have analysed and compared our results with these studies as summarized in Table 5. Comparing STNx+MI to STNx+Sham, we observed a reduction in systolic function (LVEF, ESPVR) following myocardial injury that was accompanied by a significant increase in interstitial cardiac fibrosis and hypertrophy, and tubulointerstitial renal fibrosis. These features recapitulate the renocardiac phenotype that occurs clinically. Bongartz *et al*. (2012) similarly reported a reduction in LVEF and an increase in glomerulsclerosis but did not observe any change in cardiac fibrosis, myocyte hypertrophy or tubulointerstitial fibrosis^10^. However, it is not clear whether perivascular fibrosis was included in their assessment of cardiac fibrosis, which could mask any interstitial changes. Furthermore, systolic blood pressure (SBP) is driver of cardiac and renal injury resulting in fibrosis and hypertrophy. Tail cuff measurements by Bongartz *et al*. (2012) showed a reduction in SBP from 160 mmHg in STNx+Sham to 120 mmHg in STNx+MI animals, whereas SBP in our study was above 200 mmHg in both STNx groups 10. This difference may explain the difference in fibrosis, and hypertrophy results between these studies. Diastolic dysfunction due to increased ventricular stiffness is associated with increased cardiac fibrosis 14. We did not observe an increase in diastolic dysfunction between STNx+MI and STNx+Sham; however, Bongartz *et al*. (2012) reported increased LVEDP and Tau, the time constant of early active diastolic LV relaxation, this may be due to the increased length of their study, 16 compared to 12 weeks. Windt *et al*. (2008) did not specifically examine cardiac function or assess structural changes in the myocardium; furthermore, comparison of STNx+MI with STNx+Sham did not result in any changes in renal functional or structural changes. All studies did not observe changes in renal function between both STNx groups (*P ≥* 0.05).

**Table 5:** Comparisons of pathophysiological changes in STNx+MI models

Measurement	The present study	Windt *et al*.^9^	Bongartz *et al*. 10
Duration of follow‐up (weeks)	12	12	16
Interval between STNx and MI surgery (weeks)	4	2	8
STNx+MI versus STNx+Sham			
Left ventricular ejection fraction	↓	N/A	↓
Left ventricular end diastolic pressure	↔	↔	↑
Tau logistic	↔	N/A	↑
Myocyte hypertrophy	↑	N/A	↔
Cardiac fibrosis	↑	N/A	↔
Blood pressure	↔	↔^a^	↓
Proteinuria	↔	↔	↔
Creatinine clearance	↔	↔^b^	↔
Direct glomerular filtration rate	↔	N/A	N/A
Glomerulosclerosis	N/A	↔	↑
Tubulointerstitial fibrosis	↑	↔	↔
STNx+MI versus Sham+MI			
Left ventricular ejection fraction	↔	N/A	↓
Left ventricular end diastolic pressure	↔	↔	↑
Tau logistic	↑	N/A	↑
Myocyte hypertrophy	↑	↑	↔
Cardiac fibrosis	↑	N/A	↔
Blood pressure	↑	↑^a^	↔
Proteinuria	↑	↑	↑
Creatinine clearance	↓	↓	↓
Direct glomerular filtration rate	↓	N/A	N/A
Glomerulosclerosis	N/A	↑	↑
Tubulointerstitial fibrosis	↑	↑	↑

a Blood pressure assessed in anesthetized animals.

b *P* = 0.05 towards a decrease in creatinine clearance.

↔ no significant change; ↓ decrease; ↑ increase; N/A not assessed.

The current study observed that changes in cardiac remodelling in STNx+MI were associated with an increase in cardiac gene expression of fibrotic and hypertrophy‐related markers cTGF, collagen I and ANP, and activation of the MAPK pathway compared to the STNx+Sham. Bongartz *et al*. (2012) did not observe structural changes in the heart and similarly gene expression of cTGF and hypertrophy‐related markers were not changed 10. Although renal structural changes were observed in our study, no further changes in renal gene expression or pathway activation were noted between these 2 STNx groups. This may be attributed to the dynamic nature of expression resulting in an earlier peak in gene and protein activation that has declined by week 12 15, 16.

Although structural renal changes did not result in further deterioration of renal function at 12 weeks, this is a limitation of the model where aggressive induction of CKD already reduces GFR where any further significant reductions in GFR post‐MI are difficult to observe. Despite maintaining renal function in STNx+MI animals, Windt *et al*. (2008) reported a reduction in renal blood flow in the remaining kidney compared to STNx+Sham animals. Renal blood flow was restored following treatment with an angiotensin converting enzyme inhibitor suggesting RAAS activation is increased in the STNx+MI group 9. Increased RAAS activation in this model may contribute to LV hypertrophy, LV dilation and progressive renal damage observed in the current study. Gene expression of RAAS components in STNx+MI in the kidney was no different when compared to STNx+Sham animals, of note these animals did exhibit a reduction in blood pressure and reduced RAAS gene expression in the heart 10.

Reduction in renal function and increased glomerulosclerosis and increased renal fibrosis in STNx+MI compared to Sham+MI animals was in unison agreement in all three studies (Table 5). Furthermore, we demonstrated increased macrophage infiltration with an elevation of TGFβ_1_, collagen IV and IL‐6 gene expression, and increased TGF‐β protein and NFκB activation, an important regulator of inflammation and fibrosis 17. CKD progression involves increased interleukin‐6 (IL‐6) synthesis, and impairment of nitric oxide (NO) production 18, 19. Suppression of nitric oxide metabolite excretion is thought to involve inflammatory mechanisms leading to pathological abnormalities in the remaining kidney 10. With regard to the cardiac effects between STNx+MI and Sham+MI animals, Bongartz *et al*. (2012) reported a further reduction in LVEF with no change in blood pressure, myocyte hypertrophy or fibrosis 10. In contrast, we found no change in systolic function, accompanied by increased blood pressure and adverse cardiac remodelling. In support of our findings, Windt *et al*. (2008) demonstrated increased hypertrophy, blood pressure and cardiac fibrosis; although LVEF was not assessed the load and heart rate‐dependent measure, +dP/dt_*max*_ was unchanged 9. A difference with the study by Bongartz *et al*. (2012), which may partially explain these results, is their use of Lewis inbred rats 10. While known to have a reduced mortality post‐MI and a more uniform infarct size 20, this strain does exerts reduced myocyte cross‐sectional area compared to Sprague–Dawley rats in a model of mid‐cerebral artery occlusion (MCAO) 21. The MCAO is an acute 48‐hour model, too early to detect changes in fibrosis. Another important difference between these studies is the method of STNx, Bongartz *et al*. (2012) used a two‐stage procedure where initially one kidney was removed and a week later both poles of the remaining kidney were resected, as opposed to ligation of 2/3^rd^ of the remaining kidney as performed in the current study and Windt *et al*. (2008). The latter is a more severe model of STNx compared to the two‐stage surgery regarding the effect on renal function and hypertension, observed clinically 22.

Patients with CKD have a 10‐ to 20‐fold greater risk of cardiac death compared with individuals without CKD 23. The two‐year mortality rate after MI in patients with ESRD is approximately 50% 24. Reflecting this, mortality in the current 12‐week study was 60.2% in STNx+MI compared to 22.2% in the Sham+MI group, this mortality is comparable to that obtained by Bongartz *et al*. (2012). The myocardium in STNx rats has been reported to be associated with a 15% decrease in capillary density in the heart compared to Sham animals 25, STNx+MI animals have been described to have a further 31% reduction compared to STNx 9. This could be a contributing factor towards ischaemic myocardial injury known to occur in CKD patients 26.

Clinically, it is very difficult to distinguish between chronic CRS and chronic renocardiac syndrome, often because it is not clear whether the primary cause of the syndrome is initiated by the heart or the kidney. It raises the question, is there a difference as to which is the primary failing organ? Recently, we have demonstrated that MI followed by subsequent kidney injury (MI+STNx), accelerated cardiac remodelling and renal fibrosis 12. It is therefore of interest to compare this model with the current study by pooling data from both studies (Table 6). Groups were categorized into Sham, MI, STNx, MI+STNx and STNx+MI. Similar infarct size was observed in all MI groups. MI+STNx rats displayed lower EF and increased ventricular dilatation compared to the STNx+MI group (*P* < 0.01), while a trend towards a reduction in GFR was observed in STNx+MI *versus* MI+STNx rats (*P* = 0.06). These changes occurred independent of significant SBP differences between the two groups. These findings (type 2 *versus* 4 CRS) demonstrate that animals with pre‐morbid chronic heart failure (CHF) display worse cardiac outcomes; whist those with pre‐morbid CKD may have more severe renal outcomes, suggesting disease severity in the heart or kidney reflects the primary failing organ. Although clinically, CHF and CKD are heterogeneous disorders that result from multiple underlying diseases which activate common haemodynamic, neurohormonal and immunological and/or biochemical feedback pathways 2. This culminates in a diverse and complex system in comparison with the relative simplicity of these controlled animal studies.

**Table 6:** Data pooled from MI+STNx 12 and STNx+MI (current study), with animals categorized to Sham, MI, STNx, MI+STNx and STNx+MI groups

	Sham *N* = 20	MI *N* = 18	STNx *N* = 23	MI+STNx*N* = 11	STNx+MI *N* = 7
Survival rate (%)	100.0	64.0	68.5	44.1	39.8
Infarct size (%)	–	35.2 ± 1.9	–	33.7 ± 1.5	34.4 ± 2.4
BP (mmHg)	132.1 ± 3.9	123.2 ± 4.5	221.8 ± 6.9***^,§§§^	176.6 ± 9.3§§§,###	204.9 ± 11.6§§§
LVEF (%)	67.4 ± 1.7	40.4 ± 1.4***	73.2 ± 2.0§§§	31.2 ± 1.3§,###	42.7 ± 3.5###,ªª
LV end diastolic volume (ml)	0.79 ± 0.03	1.12 ± 0.04***	0.72 ± 0.03§§§	1.23 ± 0.07###	0.97 ± 0.10§,##,ªª
LV end systolic volume (ml)	0.25 ± 0.02	0.67 ± 0.03***	0.20 ± 0.02§§§	0.85 ± 0.06§§§,###	0.55 ± 0.07§,###,ªªª
ESPVR (mmHg/μl)	0.53 ± 0.05	0.27 ± 0.03*	0.73 ± 0.09§§§	0.32 ± 0.04##	0.38 ± 0.03
EDPVR (mmHg/μl)	0.02 ± 0.01	0.04 ± 0.01*	0.04 ± 0.01**	0.04 ± 0.01	0.05 ± 0.01
Tau logistic (ms)	10.7 ± 0.4	12.6 ± 0.5**	17.1 ± 0.9***,§§§	17.0 ± 1.4§§	17.0 ± 1.6§§
LVEDP (mmHg)	3.2 ± 0.4	8.0 ± 0.8***	7.4 ± 1.0***	10.7 ± 1.5	8.8 ± 1.8
GFR (ml/min./Kg)	8.5 ± 0.3	7.4 ± 0.5	1.0 ± 0.3***,§§§	1.5 ± 0.4§§§	0.4 ± 0.3§§§,^
CrCl (ml/min.)	233.2 ± 18.8	221.6 ± 27.4	32.7 ± 6.9***,§§§	33.8 ± 7.9§§§	33.9 ± 12.4§§§
Proteinuria (mg/24 hrs)	23.9 ± 1.5	18.8 ± 1.3	366.9 ± 37.9***,§§§	433.7 ± 87.1§§§	380.0 ± 89.1§§§

Values are expressed as mean ± S.E.M.

*P < 0.05, **P < 0.01, ***P < 0.001 *versus* Sham; ^§^ *P* < 0.05, ^§§^ *P* < 0.01, ^§§^^§^ *P* < 0.001 *versus* MI; ^#^^#^ *P* < 0.01, ^#^^#^^#^ *P* < 0.001 *versus* STNx, ^ªª^ *P*<0.01, ^ªª^ ^ª^ *P*<0.001 *versus* MI+STNx.

^^^ *P* = 0.06 *versus* MI+STNx.

BP, blood pressure; LVEF, left ventricular ejection fraction; FS, fractional shortening; ESPVR and EDPVR, slope of end systolic and diastolic pressure‐volume relationship; LVEDP; LV end diastolic pressure; GFR, glomerular filtration rate; CrCl, creatinine clearance.

In conclusion, the present study has systematically examined the pathophysiology underlying renal and cardiac changes in the setting of STNx followed by MI, with critical analysis of previous studies 9, 10. In addition, the difference between chronic cardiorenal *versus* renocardiac syndromes indicates the primary failing organ results in worse outcomes for that organ when the secondary organ is significantly impaired. These findings have clinical implications with regard to the pathophysiology of chronic CRS from resultant MI in CKD patients and may represent a useful animal model for the potential assessment of mechanism‐targeted therapeutic interventions.

## Conflicts of interest

The authors declare that they have no competing financial interests.

## Supporting information


**Figure S1** Gene expression of GAPDH in cardiac tissue (A) and 18S in renal tissue (B) respectively, indicating expression of the housekeeping genes (cycle threshold Ct) were not different between the groups.
