# Supplementary figures and images for: Chronic kidney disease with comorbid cardiac dysfunction exacerbates cardiac and renal damage
Source: J Cell Mol Med. 2017 Oct 10;22(1):628–45. doi: 10.1111/jcmm.13349 (PMC5742721; doi:10.1111/jcmm.13349)

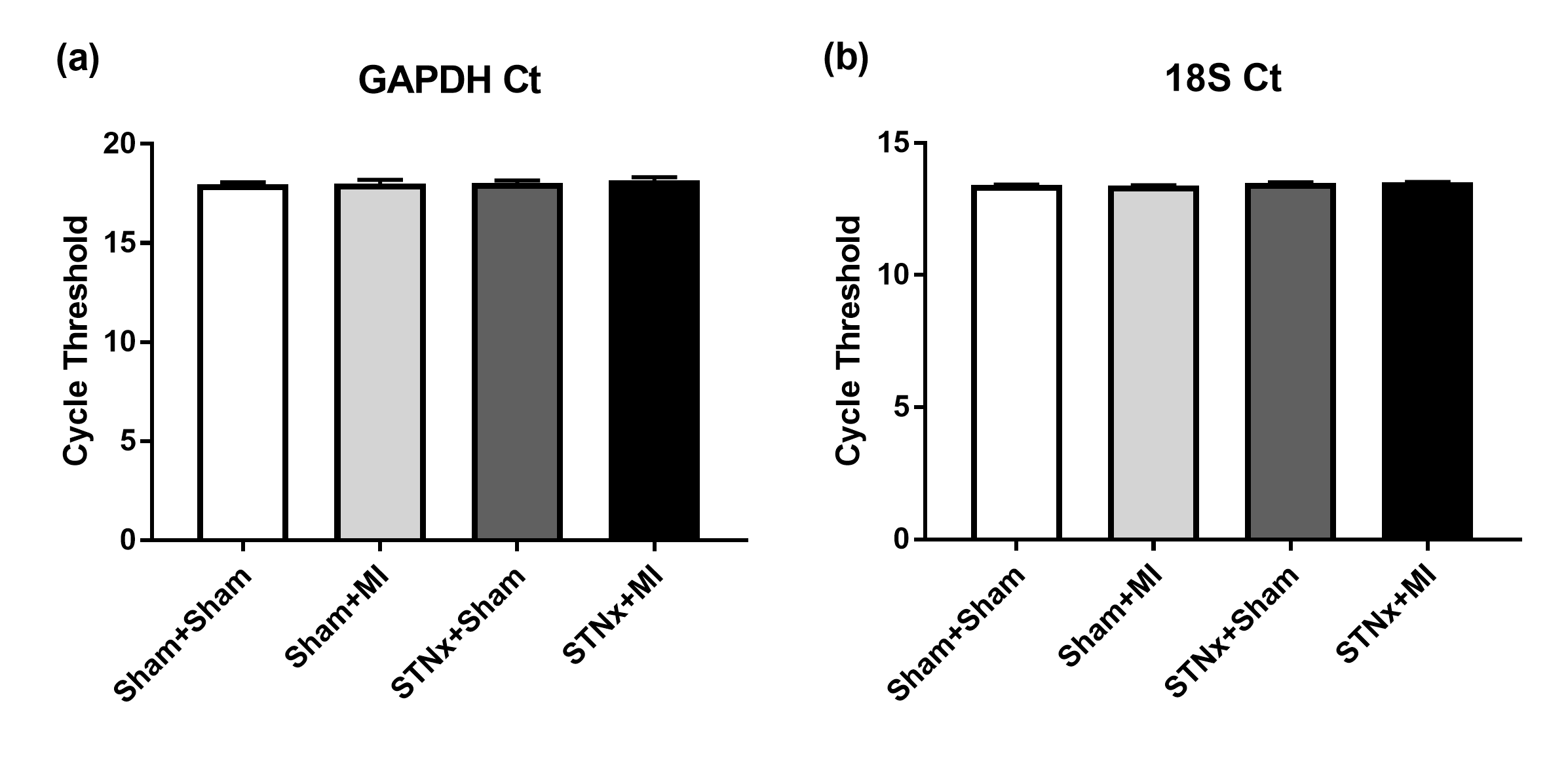

Supplement: Supplementary file 1 — Figure S1 Gene expression of GAPDH in cardiac tissue (A) and 18S in renal tissue (B) respectively, indicating expression of the housekeeping genes (cycle threshold Ct) were not different between the groups. [file JCMM-22-628-s001.tif]
